# In situ spatiotemporal measurements of the detailed azimuthal substructure of the substorm current wedge

**DOI:** 10.1002/2013JA019302

**Published:** 2014-02-12

**Authors:** C Forsyth, A N Fazakerley, I J Rae, C E J Watt, K Murphy, J A Wild, T Karlsson, R Mutel, C J Owen, R Ergun, A Masson, M Berthomier, E Donovan, H U Frey, J Matzka, C Stolle, Y Zhang

**Affiliations:** 1 Mullard Space Science Laboratory, UCLDorking, UK; 2 Department of Meteorology, University of ReadingReading, UK; 3 University of AlbertaEdmonton, Alberta, Canada; 4 Lancaster UniversityLancaster, UK; 5 Royal Institute of TechnologyStockholm, Sweden; 6 Department of Physics and Astronomy, University of IowaIowa City, Iowa, USA; 7 LASP, University of Colorado BoulderBoulder, Colorado, USA; 8 ESA/ESTECNoordwijk, Netherlands; 9 Laboratoire de Physique des Plasmas, Observatoire de Saint MaurParis, France; 10 Department of Physics and Astronomy, University of CalgaryCalgary, Alberta, Canada; 11 Space Sciences Laboratory, University of CaliforniaBerkeley, California, USA; 12 National Space Institute, Technical University of DenmarkLyngby, Denmark; 13 GFZ, German Centre for GeosciencesPotsdam, Germany; 14 John Hopkins University Applied Physics LaboratoryLaurel, Maryland, USA

**Keywords:** Substorm current wedge, Field-aligned current, Wedgelets, Aurora, Magnetosphere, Earth

## Abstract

The substorm current wedge (SCW) is a fundamental component of geomagnetic substorms. Models tend to describe the SCW as a simple line current flowing into the ionosphere toward dawn and out of the ionosphere toward dusk, linked by a westward electrojet. We use multispacecraft observations from perigee passes of the Cluster 1 and 4 spacecraft during a substorm on 15 January 2010, in conjunction with ground-based observations, to examine the spatial structuring and temporal variability of the SCW. At this time, the spacecraft traveled east-west azimuthally above the auroral region. We show that the SCW has significant azimuthal substructure on scales of 100 km at altitudes of 4000–7000 km. We identify 26 individual current sheets in the Cluster 4 data and 34 individual current sheets in the Cluster 1 data, with Cluster 1 passing through the SCW 120–240 s after Cluster 4 at 1300–2000 km higher altitude. Both spacecraft observed large-scale regions of net upward and downward field-aligned current, consistent with the large-scale characteristics of the SCW, although sheets of oppositely directed currents were observed within both regions. We show that the majority of these current sheets were closely aligned to a north-south direction, in contrast to the expected east-west orientation of the preonset aurora. Comparing our results with observations of the field-aligned current associated with bursty bulk flows (BBFs), we conclude that significant questions remain for the explanation of SCW structuring by BBF-driven “wedgelets.” Our results therefore represent constraints on future modeling and theoretical frameworks on the generation of the SCW.

**Key Points:**

1. The substorm current wedge (SCW) has significant azimuthal structure
2. Current sheets within the SCW are north-south aligned
3. The substructure of the SCW raises questions for the proposed wedgelet scenario

## 1. Introduction

The substorm current wedge (SCW) is a fundamental component of geomagnetic substorms. It represents the region in which the cross-tail current is diverted through the ionosphere; thus, it is associated with a region of dipolarized field lines which form at ∼6–10 *R*_*E*_ in the magnetotail during the substorm expansion phase [*McPherron et al.*, 1973]. This region expands radially away from the Earth and azimuthally after the substorm onset [*Lopez and Lui*, 1990; *Ohtani et al.*, 1992; *Nakamura et al.*, 2005] in association with the propagation of large-scale flapping waves in the magnetotail [*Forsyth et al.*, 2009]. The ionospheric component of the SCW gives rise to a characteristic series of deflections in the north-south, east-west, and vertical magnetic field components observed by ground-based magnetometers which is consistent with a simple line current model of the SCW [e.g., *Cramoysan et al.*, 1995; *Mann et al.*, 2008].

A number of models have been developed in order to describe the ground- and space-based observations of the SCW [e.g., *Horning et al.*, 1974; *Vasilev et al.*, 1986; *Tsyganenko*, 1987; *Sergeev et al.*, 1996; *Tsyganenko*, 1997; *Lu et al.*, 1999; *Rostoker and Friedrich*, 2005; *Sergeev et al.*, 2011]. These models generally treat the substorm current wedge as comprising of a simple line current into and out of the ionosphere and hence do not contain any more complicated cross-tail azimuthal substructure. While such models reproduce the gross large-scale structure of the substorm current wedge, as observed by azimuthally separated spacecraft and observatories, small-scale azimuthal structure which is apparent in the complex, multiscale auroral forms observed on the ground [*Borovsky*, 1993; *Sandahl et al.*, 2011] is only considered in limited studies.

Spacecraft observations have shown that the substorm time field-aligned current systems show latitudinal structuring [e.g., *Iijima and Potemra*, 1978; *Hoffman et al.*, 1985; *Fukunishi et al.*, 1993; *Hoffman et al.*, 1994]. Using data from the Triad spacecraft, *Iijima and Potemra* [1978] identified three latitudinally separated large-scale field-aligned currents in the Harang discontinuity region (2000–2400 MLT in their paper), with downward field-aligned currents bracketing a region of upward field-aligned current. Later, using data from Dynamics Explorer (DE) 2, *Hoffman et al.* [1994] subdivided these currents into seven latitudinally separated regions based on the Region 0, 1, and 2 currents identified by *Iijima and Potemra* [1976]. While the authors noted that the field-aligned current sheets could be highly tilted with respect to the average auroral oval direction, particularly in the middle of the current systems [*Hoffman et al.*, 1994], the small-scale azimuthal structure of these current systems was generally not considered. This was predominantly due to the spacecraft observations coming from polar orbiting spacecraft, such that individual crossings of the auroral regions were latitudinal rather than azimuthal.

Observationally, north-south aligned auroral forms observed in the postonset substorm auroral bulge have been shown to be related to the fast convective flow bursts in the magnetotail plasma sheet that comprise bursty bulk flows (BBFs) [*Sergeev et al.*, 2000; *Nakamura et al.*, 2005; *Forsyth et al.*, 2008]. *Sergeev et al.* [2004] showed observations of north-south auroral forms (streamers) and conjugate field-aligned currents and accompanying bursts of super-keV electron precipitation that were consistent with the pattern expected to be produced by BBFs. *Rostoker* [1991] suggested that the substorm current wedge was a combined signature of multiple small-scale features, subsequently termed “wedgelets.” It has recently been suggested that these elemental wedgelets may be driven by individual BBFs and their associated dipolarizations [e.g., *Zhang et al.*, 2011; *Lyons et al.*, 2012, 2013; *Birn and Hesse*, 2013; *Liu et al.*, 2013].

In this study, we utilize the unique multispacecraft observations of the Cluster mission during the 2010 “Auroral Acceleration Region (AAR) campaign” to probe the small- and large-scale structures of the substorm current wedge at 4000–7000 km altitude. During this AAR campaign, the spacecraft orbits were tilted away from their original polar orbits, meaning that the spacecraft crossed the auroral region moving from east to west at near constant latitudes. Using data from two of the Cluster spacecraft, we examine the spatial characteristics and temporal evolution of the substorm current wedge and compare this with observations of the magnetic field deflections on the ground. Our observations show that the SCW is azimuthally structured into a series of near north-south aligned current sheets with widths of 10-500 km, in stark contrast to the picture of east-west aligned current channels generally assumed to be linked with preonset and postonset aurora.

## 2. Observations

### 2.1. Ground Signatures of the Substorm Current Wedge

**Figure 1:**
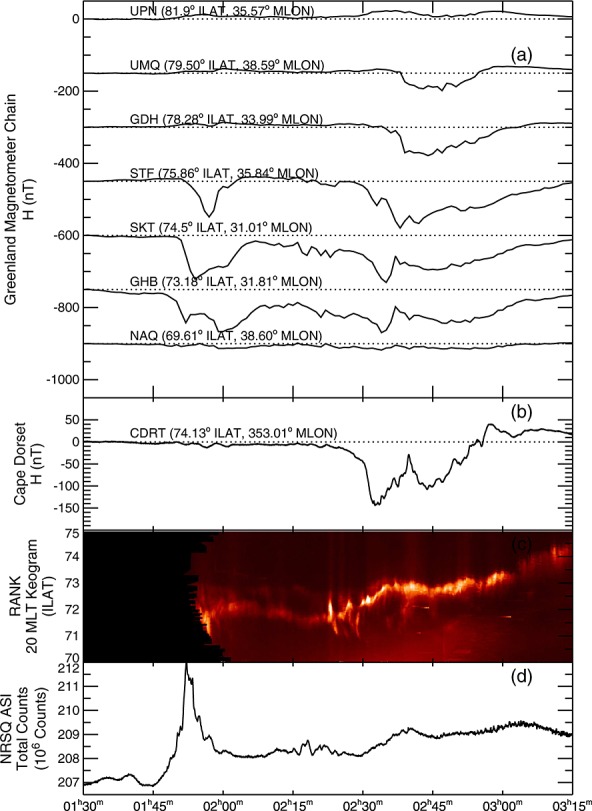
Figure showing ground magnetic field and auroral evidence of a substorm. (a) The *H* component of the ground magnetic field from the DTU magnetometer stations in western Greenland. (b) The *H* component of the ground magnetometer data from Cape Dorset. (c) Keogram of the counts at 20 MLT from the RANK all-sky imager. (d) The total number of counts from the NRSQ all-sky imager.

Magnetometer stations in Canada and Greenland detected evidence of a weak substorm between 02:15 and 03:00 UT on 15 January 2010. At this time these magnetometers covered 19-01 MLT. Figure 1a shows the *H* component of the magnetic field measured by the western Greenland magnetometer chain arranged by invariant latitude (ILAT); Figure 1b depicts the *H* component from the Cape Dorset magnetometer (CDRT). The Greenland magnetometer chain covered 31° to 38° magnetic longitude and CDRT was at 353° magnetic longitude. Figure 1c shows a keogram computed at a fixed magnetic local time (MLT) of 20:00 from the Rankin Inlet (RANK) Time History of Events and Macroscale Interactions during Substorms (THEMIS) All-Sky Imager (ASI) [*Mende et al.*, 2008], and Figure 1d shows the total counts from the Narsarsuaq (NRSQ) THEMIS ASI, which had a field of view encompassing 00:00 MLT just south of the foot points of Cluster 1 and 4. Although NRSQ did observe aurorae during this period, they appeared close to the horizon, where the mapping of auroral forms into a geomagnetic coordinate system becomes problematic, and were partially obscured by clouds. In this case, the total counts from the ASI can be used to give an indication of increasing auroral activity.

Data from the ground-based magnetometers and ASIs shows evidence of substorm activity between 01:45 and 03:00 UT. Negative magnetic bays were observed at GHB, SKT, and STF (73.18°–75.8° ILAT) between 01:45 and 02:00 UT, along with a brightening of the aurora observed at NRSQ. A further set of negative magnetic bays were observed at CDRT, GHB, SKT, and STF (73.18°–75. 8°ILAT) after 02:15 UT (Figures 1a and 1b) and subsequently expanded poleward to UMQ (79.6° ILAT), indicating a strengthening and expansion of the westward auroral electrojet. Between 02:15 and 02:30 UT, the aurora observed by the RANK ASI brightened and subsequently expanded poleward (Figure 1c). At 02:30 UT, the aurora brightened further and continued to expand poleward. This poleward expansion of the aurora was accompanied by an increase in the total counts from the NRSQ ASI (69.6° ILAT), a strengthening in the magnetic bay at CDRT (74.1° ILAT) by ∼100 nT and the formation of magnetic bays up to UMQ (79.5° ILAT). By 02:39 UT, the poleward expansion and brightening of the aurora at 20 MLT had ceased and the magnetic bays were weakening. The lack of usable auroral images over the majority of the region in question means that we cannot conclusively identify the substorm onset nor that aurora observations from RANK are of the auroral bulge. However, the poleward motion of the aurora and the negative bays in the ground magnetometer data are indicative of substorm activity during this interval, and below we provide evidence that the SCW was present during this interval.

**Figure 2:**
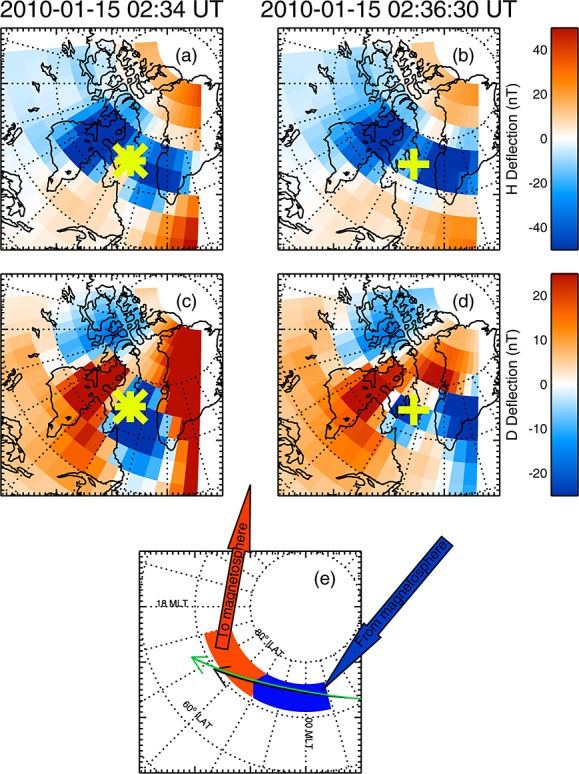
Figure showing the locations of the ground magnetic field perturbations due to the SCW calculated using the technique of *Murphy et al.* [2012] in MLT and invariant latitude coordinates. (a and b) The deflection of the *H* component of the magnetic field at 02:34 and 02:36:30 UT. (c and d) show the deflection in the *D* component of the magnetic field at 02:34 and 02:36:30 UT. The asterisks show the magnetic foot point of Cluster 4 at 02:34 UT, and the crosses show the magnetic foot point of Cluster 1 at 02:36:30 UT. (e) A summary of the interpretation of the currents from the magnetic data in terms of a simple line current model of the substorm current wedge. The foot point paths of Cluster 1 and 4 are shown in black and green, respectively. During this interval, the spacecraft foot points moved along the westward electrojet.

A two-dimensional map of the magnetic field perturbations due to the SCW ionospheric currents can only be estimated using a network of latitudinally and longitudinally spaced ground-based magnetometers. For an accurate determination of the SCW currents, a baseline quiet time preceding the interval in question is found. Deviations from this time are assumed to represent deviations due to new currents systems or enhancements to existing current systems. For this event we use a baseline time of 02:15 UT. The three components of the magnetometer data set are interpolated onto a constant spatial grid to generate a map of magnetic field perturbations as a function of time [*Murphy et al.*, 2012]. In this study we use data from the following magnetometer arrays: Canadian Array for Realtime Investigations of Magnetic Activity (CARISMA) [*Mann et al.*, 2008], THEMIS ground-based observatories and education and outreach program [*Russell et al.*, 2008; *Peticolas et al.*, 2008], midcontinent magnetoseismic chain [*Chi et al.*, 2013], Magnetometer Array for Cusp and Cleft Studies [*Engebretson et al.*, 1995], INTERMAGNET (http:\www.intermanget.org), and Greenland magnetometer chain (http://www.space.dtu.dk/English/Research/Scientific_data_and_models/Magnetic_Ground_Stations.aspx). Figures 2a and 2b show maps of the deflection of the *H* components of the magnetic field, and Figures 2c and 2d show the *D* components of the magnetic field at 02:34 (Figures 2a and 2c) and 02:36:30 UT (Figures 2b and 2d). These maps are presented on a grid of MLT and invariant latitude. Figure 2(green crosses) shows the foot points of C1, and Figure 2(green asterisks) show the foot points of C3 at the times of the plots, showing that the spacecraft foot points moved along the westward electrojet.

Simple line current models of the SCW predict that the deflection in the *H* component of the ground magnetic field is negative along the auroral electrojet and positive north and south of it [*Cramoysan et al.*, 1995; *Mann et al.*, 2008]; thus, the location of the auroral electrojet can be identified as an east-west band of negatively deflected *H* component. Such a signature is shown in Figures 2a and 2b, with a negative (blue) band between 20:00 and 01:00 MLT between 70° and 80° ILAT. These models of a pair of field-aligned currents (FACs) in the SCW also predict that the deflection in the *D* component forms a quadrupolar pattern in which one inversion line lies along the auroral electrojet and the other indicates the midplane of the ionospheric signature SCW. Figures 2c and 2d show this quadrupolar pattern and place the midplane of the SCW at ∼22:00 MLT. The inferred current system from the ground-based magnetometers is summarized in Figure 2e, which shows the approximate locations of the upward (red) and downward (blue) currents (assuming a simple line current model) and the auroral electrojet.

Taken together, these data show that occurrence of a substorm after 01:45 UT and encompassing the time of the Cluster crossing. The SCW spanned 5 h of MLT and the auroral electrojet lay between 70° and 80° ILAT. The substorm was small, with a maximum deflection in the *H* component of the ground magnetic field of 150 nT and a 0.5°–1° contraction of the auroral oval over RANK.

### 2.2. Cluster Observations of the Substorm Current Wedge

Changes in the Cluster orbit mean that between 2007 and 2013 the spacecraft passed over auroral latitudes at altitudes as low as 3000 km [*Forsyth and Fazakerley*, 2012]. A subset of these orbits “skimmed” the auroral oval, crossing several hours of MLT at near constant latitude. The Cluster Northern Hemisphere auroral crossing on 15 January 2010 was one such orbit. During the perigee pass of Cluster tetrahedron on this day, data were available from Cluster 1, 2, and 4 but not from Cluster 3.

**Figure 3:**
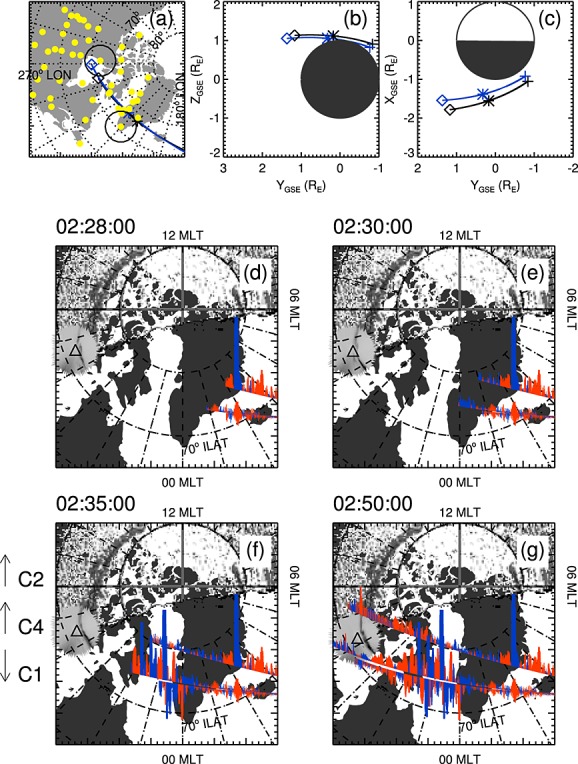
Figure showing the locations of Cluster 1 (black), Cluster 4 (blue), and the ground-based instrumentation used in this study. (a) The spacecraft foot points in geographic latitude and longitude. The crosses and diamonds show the spacecraft locations at 02:30 and 02:45 UT, respectively. The underlying map shows the locations of the ground magnetometer stations (yellow dots) and the fields of view of the RANK and NRSQ all-sky imagers at 02:30 UT. (b and c) The locations of Cluster 1 and Cluster 4 projected on the *YZ* and *YX* GSE planes, respectively. (d–g) The spacecraft foot point tracks from Cluster 1, Cluster 2, and Cluster 4 up to 02:28, 02:30, 02:35, and 02:50 UT. Overlaid on the spacecraft track are the magnetic field gradients perpendicular to the spacecraft track and the *Tsyganenko and Stern* [1996] magnetic field (negative gradients in red, positive gradients in blue). Gradients from Cluster 1 point down the page, and gradients form Cluster 2 and Cluster 4 point up the page. The dayside auroral data are from a single image compiled from DMSP SSUSI data from around 02:40 UT. Also plotted are auroral data from the THEMIS ASI at RANK projected to 110 km altitude. Auroral data are plotted in gray scale with darker colors indicating brighter aurora. An animation incorporating Figures 3d–g is provided in the supporting information.

Figure 3a shows the magnetic foot points in geographic latitude and longitude of Cluster 1 (black) and Cluster 4 (blue) along with the locations of magnetometer stations in Canada [*Engebretson et al.*, 1995; *Mann et al.*, 2008; *Russell et al.*, 2008; *Peticolas et al.*, 2008] and Greenland (http://www.space.dtu.dk/English/Research/Scientific_data_and_models/Magnetic_Ground_Stations) along with the fields of view of the THEMIS ASIs at RANK and NRSQ [*Mende et al.*, 2008]. Figures 3b and 3c show the spacecraft locations projected on the GSE *YZ* and *YX* planes, respectively. In order to put the spacecraft locations in context, Figures 3d–3g show the spacecraft foot points of Cluster 1, 2, and 4 up to 02:28, 02:30, 02:35, and 02:50 UT, respectively, in MLT and ILAT coordinates. Projecting from the spacecraft foot point paths are the magnitudes of the gradients in the magnetic field perpendicular to the background (T96) [*Tsyganenko and Stern*, 1996] field and the spacecraft trajectory, corresponding to upward (red) and downward (blue) field-aligned currents. The gradients are calculated from data from the fluxgate magnetometers (FGM) [*Balogh et al.*, 2001] on the spacecraft. Magnetic field gradients shown in Figures 3d–3g from Cluster 2 and Cluster 4 point up the page and magnetic field gradients from Cluster 1 point down the page. The dayside auroral data were obtained around 02:40 UT from a single FUV (N2 Lyman-Birge-Hopfield band short: 140–150 nm) image compiled from F16 Defense Meteorological Satellite Program (DMSP) Special Sensor Ultraviolet Scanning Imager (SSUSI) [*Paxton et al.*, 2002; *Zhang et al.*, 2008]. Also shown is the white light aurora observed by the THEMIS ASI at RANK projected to an altitude of 110 km. Auroral brightness is grey scaled such that darker grey corresponds to more intense auroral emission.

Cluster 1 and Cluster 4 passed over Greenland at approximately 02:30 UT and altitudes of 6300 and 4900 km, respectively, within the expected altitude range of the quasi-static auroral acceleration region [*Lindqvist and Marklund*, 1990; *Lu et al.*, 1992; *Reiff et al.*, 1993]. The spacecraft moved westward, crossing 3 h of magnetic local time (MLT) in 12 min and 8 min, respectively. The spacecraft foot points remained above 70° ILAT and between 02:00 MLT and 20:00 MLT and followed approximately the same path. Cluster 1 was 1300–2000 km higher than Cluster 4 at the same magnetic local time, but Cluster 4 led Cluster 1 by 143 s at 00:00 MLT, increasing to 238 s by the time Cluster 1 reached 22:00 MLT.

Cluster 1 and 4 observed a region of upward field-aligned current at 71°–73° ILAT between 02:00 and 01:30 MLT. This upward current system was stable, being observed in the same location and with the same strength by both spacecraft. Subsequently, the spacecraft moved into a region in which the magnetic field gradients were small (01:30 to 00:00 MLT). Between 00:00 and 22:30 MLT Cluster 1 and Cluster 4 detected magnetic field gradients associated with upward and downward field-aligned current (FAC) that were much larger than those detected as the spacecraft crossed the postmidnight auroral oval. In contrast to the currents observed between 02:00 to 01:30 MLT, the magnetic field gradients observed at the same magnetic foot point location (but different times) by Cluster 4 and Cluster 1 differed in both magnitude and direction. Cluster 1 and 4 exited the region of large magnetic field gradients at ∼73° ILAT, consistent with a continued projection of the aurora observed by the RANK ASI.

For context, Cluster 2 observed distinct upward current regions between 72° and 75° at 02:45 MLT and 19:00 MLT. The currents observed at 19:00 MLT coincided with the auroral arc observed by the RANK ASI; hence, it is likely that Cluster 2 was on auroral field lines at these times. The magnetic field gradients between these times were relatively small, although there is a distinct pattern suggesting the presence of net downward current west of 22:00 MLT and net upward current east of 22:00 MLT. Data from the Plasma Electron And Current Experiment (PEACE) [*Johnstone et al.*, 1997] instrument (not shown) shows a drop in electron flux above 32 eV, showing that Cluster 2 passed through the polar cap between 02:00 and 19:20 MLT (02:28 UT and 02:44 UT).

Given that Cluster 2 was in the polar cap, and the unavailability of data from Cluster 3, we limit this study to using data from Cluster 1 and 4. Under these restrictions, we are unable to determine current densities using the multispacecraft curlometer technique [*Dunlop et al.*, 1988; *Robert et al.*, 1998] but instead use a single spacecraft technique (described in Appendix A).

**Figure 4:**
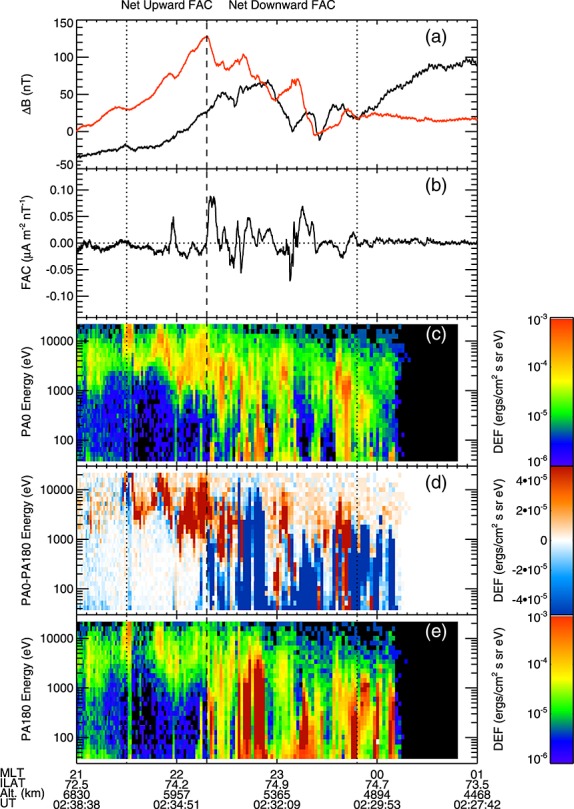
Figure showing data from the Cluster 4 crossing of the substorm current wedge. (a) The residual magnetic field (with the *Tsyganenko and Stern*[1996] model removed) perpendicular to the model field in the northward (black) and westward (red) directions. (b) The field-aligned current density calculated using a single spacecraft method (negative in the direction away from the Earth, see Appendix A). (c–e) The electron differential energy flux in the field parallel direction (Figure 4c), the field anti-parallel direction (Figure 4e), and the difference in differential energy flux (Figure 4d) between the field parallel and field antiparallel directions (red toward the Earth). The data are plotted against the magnetic local time of the spacecraft foot point. The dashed line shows the boundary between the net downward and net upward FAC regions, and the dotted lines show the edges of these regions.

**Figure 5:**
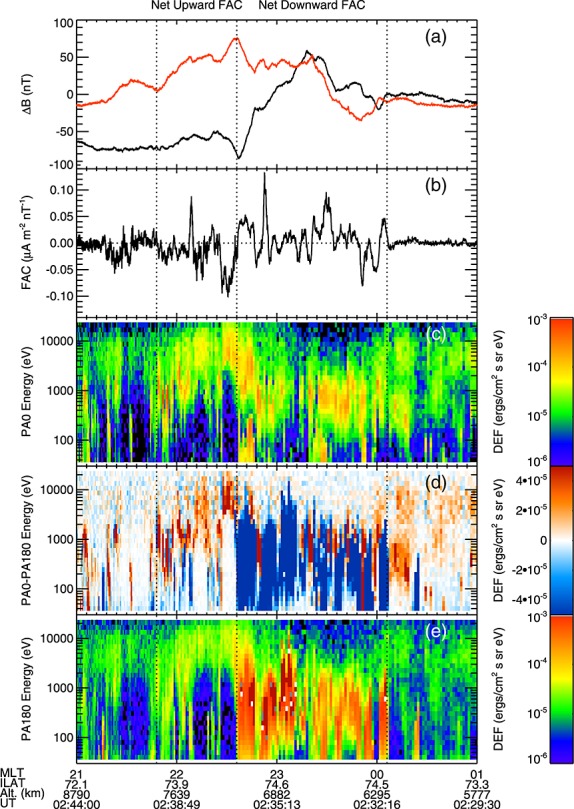
Data from Cluster 1 presented in the same format as Figure 4.

Figures 4a and 5a show the residual magnetic field perpendicular to the T96 model magnetic field in the northward (red) and westward (black) directions; Figures 4b and 5b show the field-aligned current densities per unit magnetic field calculated from the magnetic field data using a single spacecraft method (see Appendix A); Figures 4c–4e and 5c–5e show the PEACE electron differential energy flux spectra in the downward (Figures 4c and 5c) and upward (Figures 4e and 5e) field-aligned directions and the difference in differential energy flux between the downward and upward directions (Figures 4d and 5d). By using the field-aligned current density per unit magnetic field, we can directly compare the currents observed by Cluster 4 and Cluster 1 accounting for the decreasing area of a flux tube with height under the assumption ∇.**j**=0 [*Forsyth et al.*, 2012]. These data are plotted against the MLT of the spacecraft foot points.

Cluster 4 passed into the auroral region 140 s before Cluster 1 and 1400 km lower in altitude. Cluster 4 observed a region of net downward (positive) field-aligned current between 23:48 and 22:18 MLT (Figure 4b), characterized by a generally increasing trend in the northward component of the residual magnetic field (Figure 4a) and predominantly upward electron energy flux (Figure 4d, blue). We note that Cluster was moving from east to west during this time (i.e., to earlier MLT); thus, universal time runs opposite to MLT in Figures 4 and 5. An examination of the electron distribution functions in the downward current regions showed that the electron populations were hot and moving upward along the magnetic field, consistent with previous observations of downward current regions [*Paschmann et al.*, 2003, and references therein].

Duskward of the region of net downward FAC, between 22:18 and 21:30 MLT, Cluster 4 passed through a region of net upward negative FAC, characterized by a generally decreasing trend in the northward component of the residual magnetic field (Figure 4a) and predominantly downward electron energy flux (Figure 4d, red). The electron energy flux peaks were between 1 and 10 keV, and the electron distribution functions showed the presence of an electron population accelerated downward, parallel to the magnetic field, indicative of the spacecraft being within or below upward current AARs [*Paschmann et al.*, 2003, and references therein].

The data from Cluster confirms the large-scale currents determined by the ground-based magnetometer data (Figure 2), showing regions of net upward and downward field-aligned current. However, the Cluster data also show that these regions showed structuring that was not revealed by the ground magnetometer data. Magnetic field data from Cluster 4 (Figures 4a and 4b) show that within the broad regions of net downward and upward field-aligned current, there were relatively small regions of oppositely directed current (i.e., upward current in the net downward current region and vice versa). This is echoed in the electron data, for example with regions of downward field-aligned electrons observed in the net downward current region (Figure 4d, red).

Data from Cluster 1 show a number of similarities to that from Cluster 4. The large-scale net downward and upward current regions can be identified, both from the magnetometer data (Figures 5a and 5b) and from the difference in the electron energy flux (Figure 5d), although both these regions were displaced by ∼16 min MLT toward dawn. The net downward electron energy flux in the downward region extended to higher energies at Cluster 1, and the net upward electron energy flux in the upward region was weaker at Cluster 1 than at Cluster 4.

**Figure 6:**
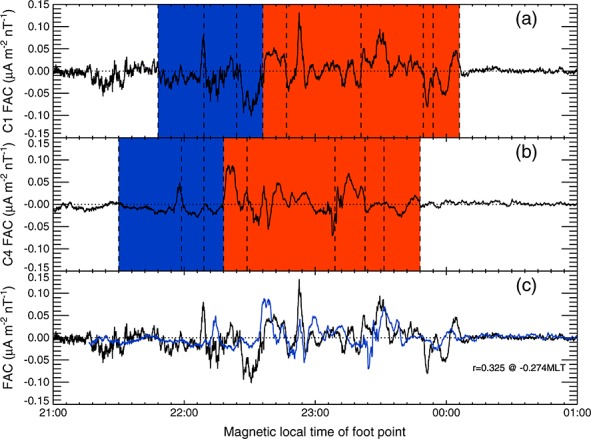
Figure showing a comparison of the field-aligned current density per unit magnetic field plotted against magnetic local time of the spacecraft foot points from (a) Cluster 1 and (b) Cluster 4. (c) The same data overlaid on the same trace with the Cluster 4 data shifted by 0.274 MLT. The blue and red shaded regions in Figures 6a and 6b show the net downward and upward current regions, respectively. The dashed vertical lines indicate mesoscale current regions which were identified in each data set.

Examining the currents observed by Cluster 4 and Cluster 1 by eye, we can find a number of features in the two time series that we interpret as the same mesoscale current sheets drifting apart or together as a function of time. Figure 6 shows the FAC density per unit magnetic field from (Figure 6a) Cluster 1 and (Figure 6b) Cluster 4, with eight mesoscale regions that are delimited by dashed lines. Figure 6c shows the Cluster 1 currents in black overlaid by the Cluster 4 currents in blue, with the Cluster 4 currents having been shifted by −00:16:30 MLT, determined as the MLT shift which gives the maximum cross correlation between the two data sets.

The linear cross correlation between the field-aligned current data from the two spacecraft was poor (*r*=0.325); however, we interpret this as being due to temporal variations of the currents. Comparing the individual mesoscale current regions shows that the variations in the current sheets were nonuniform. The upward current sheets close to 22:00 MLT moved eastward by 16 min MLT but had approximately the same widths during the two crossings, whereas the upward currents at the eastern edge (00:00 MLT when observed by Cluster 1) moved and narrowed in MLT. The currents observed by Cluster 1 in the net upward region were stronger, suggesting an increase in the current strength over time but in the net downward current region the currents observed by Cluster 1 and Cluster 4 were comparable.

**Figure 7:**
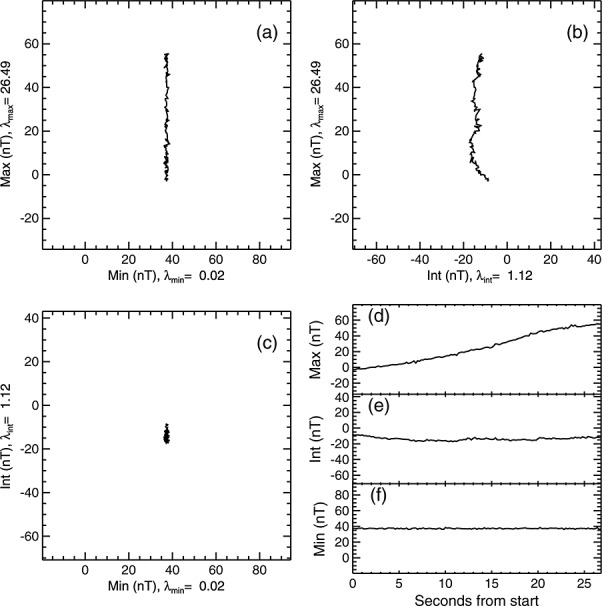
An exemplar hodogram of the results of MVA on a current sheet crossing by Cluster 1. The data interval for this analysis is 02:33:25–02:33:52 UT. (a) The maximum variance component versus the minimum variance component, (b) the maximum variance component versus the intermediate variance component, and (c) the intermediate component versus the minimum variance component. (d–f) The maximum, intermediate, and minimum variance components plotted against seconds from the start of the crossing. The ratio between the maximum and intermediate Eigenvalues was 23.7 and between the intermediate and minimum Eigenvalues was 56, indicating that all the variance directions were well defined.

Minimum variance analysis (MVA) [*Sonnerup and Cahill Jr*., 1967; *Sonnerup and Scheible*, 1998] can be used to determine the orientation of the individual current sheets that make up the substructure of the SCW. From the magnetic field gradients we identified 26 small-scale current sheets in the Cluster 4 data and 34 small-scale current sheets in the Cluster 1 data. Figure 7 shows a hodogram of an exemplar current sheet crossing, showing (a) the maximum versus minimum components, (b) the maximum versus intermediate components, (c) the intermediate versus minimum components and time series plots of (d) the maximum, (e) intermediate, and (f) minimum components. The hodogram shows that, for this current sheet, the field variation was almost entirely contained within the maximum variance direction, as one would expect for a current sheet that is essentially infinite in two directions. The maximum-intermediate Eigenvalue ratio was greater than 5 for 56 of the identified current sheets, indicating that the maximum variance direction was well defined in nearly all cases.

**Figure 8:**
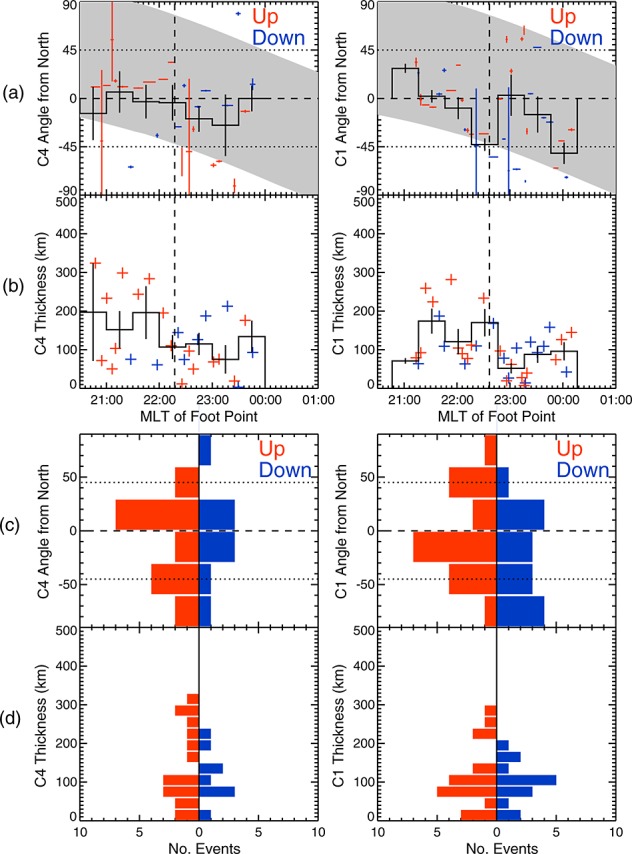
Figure showing the orientation and thicknesses of the individual current sheets within the SCW. The orientation of the current sheet is the angle between the maximum variance direction and the northward direction perpendicular to the magnetic field. Data from Cluster 4 are shown in the left hand column, and data from Cluster 1 are shown in the right hand column. (a) Row shows the orientation of upward (red) and downward (blue) current sheets against the MLT of the spacecraft foot point. Uneven crosses are used to show the MLT range covered by each current sheet and the uncertainty in the current sheet orientation. This uncertainty was calculated using a bootstrap technique [*Kawano and Higuchi*, 1995; *Sonnerup and Scheible*, 1998]. The grey shaded area shows those angles which are more than 30° away from the spacecraft trajectory; thus, the FAC density can be corrected for the current sheet orientation (see Appendix A. (b) Row shows the thickness of the current sheets, corrected for their orientation. The vertical dashed lines in rows of Figures 8a and 8b show the boundary between the net upward and net downward current regions, with the net upward region duskward of the boundary. The black lines in rows of Figures 8a and 8b show the mean values in 30 min MLT bins, with the standard error plotted as vertical black bars. (c and d) Rows show histograms of the orientation and thickness of the upward (red) and downward (blue) current sheets.

Figure 8 shows the orientations and thicknesses of the individual current sheets as calculated using MVA (see Appendix A). In Figures 8a–8d, red indicates upward current sheets and blue indicates downward current sheets. Figure 8a shows the angle of the current sheets away from the northward direction plotted against MLT for Cluster 4 and Cluster 1 (Figure 8a, right). Uneven crosses are used to show the MLT width of the current sheet (along the spacecraft track) and the uncertainty of the angle. The uncertainty is calculated from a bootstrap method to draw random vectors from the original data set and recalculate the MVA directions, repeating this 500 times [*Kawano and Higuchi*, 1995; *Sonnerup and Scheible*, 1998]. The uncertainty is then given as the standard deviation of the calculated angles. Figure 8 (grey shaded area) shows those current sheets whose orientation was more than 30° away from the spacecraft trajectory, such that we could reliably adjust the calculated FAC density to account for the nonnormal crossing of the current sheet by the spacecraft (see Appendix A).

Figure 8b shows the thicknesses of the current sheets against MLT, corrected for the angle between the current sheet and the spacecraft trajectory. In Figures 8b, even crosses are used. In Figures 8a and 8b, the vertical line indicates the boundary between the net upward current and net downward current regions. Figures 8c and 8d show histograms of the orientation of the current and the thicknesses of the sheet, respectively.

Figures 8a and 8b (black traces) show the mean values in bins of 30 min of MLT, with the vertical black bars indicating the standard error in the means. The bins used for the C4 data have been shifted by 16 min MLT to account for the dawnward drift of the current sheets.

The current sheets observed by Cluster 4 were generally within ±30° of the north-south direction (Figures 8a and 8c), although we note that a small number of current sheets can be seen in all directions including parallel to the satellite tracks (in the east-west direction). In particular, Figure 8a shows that in the net downward current region the downward current sheets were close to north-south aligned and in the upward current region the upward current sheets were close to north-south aligned. In contrast, the opposite sense currents (i.e., upward current sheets in the net downward current region) were less well aligned with the north-south direction, leading to the peak in the upward current sheet orientations between −30° and −60° in the Cluster 4 data in Figure 8c.

The orientations of the current sheets observed by Cluster 1 were less well ordered, particularly in the net downward current region. The Cluster 1 data in Figure 8c shows an almost uniform distribution of downward current sheet orientations across the 30° degree bins between +30° and −90°. The orientations of the upward current sheets still peaked within 30° of the north-south direction. Comparing the Cluster 1 data in Figures 8a and 8c show that the orientations of the currents sheets had a greater spread of values in the net downward current region but were within ±45° of the north-south direction in the upward current region.

The distribution of the thicknesses of the current sheets were similar between the Cluster 4 and Cluster 1 crossings. Both the upward and downward current sheet thicknesses peaked at 100 km (Figure 8d), which maps down to ∼50 km in the ionosphere. The range of thicknesses of the downward current sheets was up to 200 km for both the Cluster 4 and Cluster 1 crossings, whereas the thicknesses of the upward current sheets extended up to 300 km. Figure 8b shows that current sheets up to 200 km thick were observed in both the net upward and net downward current regions. The wide (200 km to 300 km) sheets were only upward current sheets and were only observed in the net upward current region.

During the relatively short time between the two spacecraft crossings, the data show that the orientation of the current sheets became less well ordered, particularly in the downward current region. Assuming that some of the current sheets observed by Cluster 4 and were also observed by Cluster 1, we can find no systematic change between the two crossings, implying that each of the current sheets varied independently but that this variability was more constrained within the net upward current region. Using the Student's T-test, we found that there was no significant difference in the means of the current sheet orientations of sheet thicknesses between the two crossings. The exceptions to this were the current sheet orientations in the 23:30–00:00 MLT bin (23:46–00:16 MLT for C1) and the current sheet thicknesses in the 22:30–23:00 MLT bin (22:46–23:16 MLT for C1).

**Figure 9:**
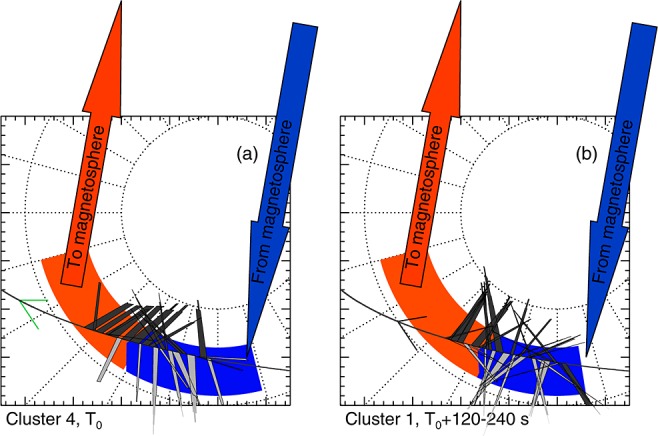
Figure showing a summary of the observations of the SCW in invariant latitude and magnetic local time coordinates. (a) The current magnitudes and orientations from Cluster 1 and (b) the current magnitudes and orientations from Cluster 4. Upward currents are shown in dark grey and point up the page, downward currents are shown in light grey and point down the page. Also shown is the location of the auroral electrojet with blue indicating those regions associated with the downward current region and red showing those regions associated with the upward current region (after Figure 2).

Figure 9 shows a summary of our observations of the substorm current wedge from Cluster compared with the locations of the magnetic disturbance on the ground. The observations from Cluster 4 and Cluster 1 indicate that the currents within the substorm current wedge were highly structured in the azimuthal direction. On the largest scale, the results are consistent with the observations from the ground, with a region of net downward current between ∼22 and 00 MLT and a region of net upward current between ∼20 and 22 MLT. However, these downward and upward current regions contained a plethora of smaller-scale current sheets, with upward and downward currents apparent in both regions. In the upward current region, the upward current sheets were close to north-south aligned and were thicker. In the downward current region, the downward currents were initially north-south aligned but in under 240 s had rotated to be less well ordered and had a greater range of orientations.

## 3. Discussion

Historically, the substorm current wedge has been idealized as a simple line current flowing into the ionosphere at the eastern edge of the substorm auroral bulge and out of the ionosphere through the auroral surge head at the western edge of the auroral bulge, with a westward electrojet closing these two FACs. The FACs bracket a region of dipolarized magnetic field in the magnetosphere [*McPherron et al.*, 1973, and references therein]. This picture is supported by large-scale ground-based observations [*McPherron et al.*, 1973] and highly separated space-based observations [e.g., *Walsh et al.*, 2010]. While some models of the SCW now include additional current loops at the low-latitude edge of the SCW [*Sergeev et al.*, 2011] or suggest that existing equivalent current systems move in local time [*Rostoker and Friedrich*, 2005], they still do not fully consider the azimuthal structure of the SCW. Recently, *Murphy et al.* [2013] showed that currents in the substorm current wedge observed by AMPERE [*Anderson et al.*, 2000; *Waters et al.*, 2001] were structured at ∼MLT and ∼1° ILAT scales. When the currents in these structures are summed over a larger area, the net currents return the traditional large-scale SCW pattern. In this study, we have shown that the azimuthal structuring of the SCW extends to even smaller scales and that the alignment of the observed current sheets within the SCW was more strongly north-south than east-west. We note, however, that as both spacecraft followed the same ground track through the SCW, we cannot determine the presence of east-west oriented currents away from the spacecraft locations. The data presented from ground-based magnetometers in Greenland and Canada exhibited current signatures associated with the SCW that reflected the traditional simple line current model (c.f. Figure 2). Given that ground-based magnetometers detect the integrated effects of all ionospheric currents, through the Biot-Savart law, and are ∼100 km separated from the ionospheric current systems, they cannot determine the effect of small-scale currents observed by the Cluster spacecraft, which passed directly through the currents. When the small-scale currents observed by Cluster are integrated over a sufficiently large path, the large-scale current system observed on the ground is recovered as shown by the FGM data (Figures 4a and 5a).

The closure of the auroral current system and the electrodynamics associated with the substorm current wedge have previously been shown to be more complex and localized than the simple line current model suggests [*Marklund et al.*, 1998, 2001; *Amm and Fujii*, 2008]. In particular, the upward currents associated with the auroral surge, taken to be the ionospheric foot point of the western edge of the substorm current wedge, have been shown to close locally to the surge [*Marklund et al.*, 1998], with only 30–40% of the upward currents in the surge transmitted along the Cowling channel to be closed remotely [*Amm and Fujii*, 2008]. Our observations show that as Cluster 1 and 4 crossed the substorm current wedge, they passed through regions of net downward and net upward current but that oppositely directed currents were detected in these regions, suggesting some localized current closure. In the net downward current region, we estimate that 42–47% of the downward FAC were accounted for by the upward FAC. In the net upward field-aligned current region, we estimate that 18–25% of the upward FAC were accounted for by the downward FAC. This suggests that there may be greater local current closure in the net downward current region. It is important to note, however, that the Cluster spacecraft were passing east-west through the substorm current wedge; thus, we can only investigate local current closure in that direction and not to the north, thereby missing important current closure near the auroral surge [*Marklund et al.*, 1998; *Amm and Fujii*, 2008; *Murphy et al.*, 2013].

The apparent north-south alignment of the currents observed in the upward current region and in the Cluster 4 crossing of the downward current region are in contrast with previous statistical analysis of auroral currents by *Hoffman et al.* [1994]; *Peria et al.* [2000] who showed that auroral current sheets tend to be oriented east-west, but in keeping with observations of north-south aligned auroral forms in the substorm bulge [e.g., *Rostoker and Eastman*, 1987; *Henderson et al.*, 1998; *Lyons et al.*, 1999; *Sergeev et al.*, 2004; *Forsyth et al.*, 2008]. *Hoffman et al.* [1994] (subsequently modeled by *Gjerloev and Hoffman* [2002]) examined 39 substorm time crossings of the auroral oval during by Dynamics Explorer (DE) 2, based six longitudinal sectors (defined with respect to the auroral bulge) and into different current regions (e.g., Region 0, 1, and 2 currents and mixed current regions); thus, they did not necessarily examine individual current sheets. They found that across the bulge the Region 0, 1, and 2 currents were aligned with L shells (to with ±20°) and that currents in the mixed region (between Region 1 and 2) showed greater inclination, although they state that they did not have evidence of the spacecraft passing through north-south aligned auroral fingers. *Hoffman et al.* [1994] also found that the mixed region made up between 23 and 52% of the width of the auroral bulge *Peria et al.* [2000] used an automated routine to detect current sheet crossings by FAST and determine their orientation. They found that the auroral current sheets were predominantly east-west aligned, but their results did not separate out current sheet crossings under different geomagnetic conditions. Studies using Freja [*Frey et al.*, 1998], DMSP [*Sergeev et al.*, 2004], and Cluster in the auroral region have shown that both north-south aligned and east-west aligned features can be observed [*Sadeghi et al.*, 2011], that these features can rotate between spacecraft crossings on the time scale of a few minutes [*Forsyth et al.*, 2012], and that the orientation of the current sheets may be dependent on latitude [*Frey et al.*, 1998].

The use of single spacecraft magnetic field data to determine the orientations of currents does introduce some bias with the direction of finite length current sheets that are closely aligned with the spacecraft trajectory being poorly defined. DE-2 and FAST both had orbits that were close to north-south aligned, such that the results of *Hoffman et al.* [1994] and *Peria et al.* [2000] are biased toward east-west aligned currents. The trajectory of the Cluster spacecraft in this study varied from being predominantly east-west near 23:00 MLT to being more north-south aligned at 01:00 MLT and 21:00 MLT, as indicated by the grey regions in Figure 8a. The data from C4 in Figure 8a also shows that, despite the variation in the spacecraft's trajectory with respect to the north-south direction, the orientation of the current sheets remains approximately constant, suggesting that these current sheet orientations are well determined. Furthermore, Figure 8a shows that a full range of current sheet orientations were observed during the spacecraft crossings; hence, we believe any biasing of the current sheet orientation related to the trajectory of the spacecraft relative to the current sheet is minimized.

From in situ observations of the magnetic field, we cannot determine the orientation of currents sheets away from the spacecraft's location. As such, there may have been east-west aligned current sheets that were not observed by Cluster such that we cannot determine what fraction of the SCW is made up of north-south or east-west aligned currents. However, the results of the MVA on the magnetic field data indicates that the field-aligned currents formed current sheets; therefore, the sheets must have a minimum length comparable to their width; 100 to 300 km corresponding to ∼1 to 3° ILAT. Figure 2 shows that the auroral electrojets extended over 10°±5° ILAT; thus, it is possible that the current sheets extended across much of the SCW. We note that *Hoffman et al.* [1994] found that the “mixed current region,” in which they observed current sheets highly inclined to L shells, corresponded to between 7 and 52% of the total latitudinal width of the auroral currents observed.

As there were no auroral observations available at the foot points of Cluster 1 and 4, we cannot directly link the observed currents with auroral features. However, Figure 3g shows that Cluster 2 observed magnetic field gradients associated with upward FACs above an auroral arc at 19:00 MLT, and these gradients were smaller than those observed by Cluster 1 and 4 in the SCW. As such, we can infer that the FAC densities observed by Cluster 1 and 4 would have been associated with aurora and thus can compare our observations with the previous observations of various auroral forms and speculate on the likely link between the spacecraft observations and these features.

It has been suggested that the SCW may be formed of a number of “elemental components” or wedgelets and that these, in turn, are associated with bursty bulk flows (BBFs) in the magnetotail [*Rostoker*, 1991; *Lyons et al.*, 2012; *Liu et al.*, 2013]. In space, wedgelets cause localized dipolarizations of the magnetotail and are coupled with FACs into the ionosphere on their dawnside and out of the ionosphere on the duskside, in the same sense as the FACs in the SCW. The exact mechanism by which BBFs can make up the SCW is unclear; however, the simplest we can suggest is that the large-scale signatures are a simple addition of the signatures of each individual BBF. In the net downward current region, our observations show some similarities with this concept of multiple wedgelets in that we see a number of upward and downward currents; however, the current sheets reported in this study do appear to form distinct pairs of currents, as expected from models of BBFs as depleted flux tubes [*Chen and Wolf*, 1993, 1999; *Birn et al.*, 2004]. Furthermore, these currents show distinct differences from the in situ field-aligned currents seen in BBFs [*Forsyth et al.*, 2008] and the FACs in their ionospheric counterpart, auroral streamers [*Amm et al.*, 1999; *Nakamura et al.*, 2005; *Juusola et al.*, 2009]. These previous studies of both the ionospheric signatures and in situ observations of the FACs associated with BBFs all show that the upward FAC density was stronger and spread out over a narrower area than the downward FAC density. This pattern is consistent during substorm and nonsubstorm times [e.g., *Juusola et al.*, 2009]. In contrast, the observations presented in this study show the opposite relationship; in the net downward current region, the downward currents were generally stronger than the upward currents and in the net upward current region, the upward currents were wider than the downward currents. Furthermore, upward current sheets were not necessarily abutted by downward current sheets (and vice versa), as one might expect. This is particularly clear in the net upward current region. As such, by comparing our observations with previous observations of BBF current systems we find that our observations do not support the scenario in which BBFs create the small-scale structure comprising the SCW. We note that the occurrence of BBFs and north-south aligned auroral forms increases with substorm activity [*Angelopoulos et al.*, 1994; *Lyons et al.*, 1999; *Juusola et al.*, 2011] and multiple streamers can be observed in the substorm bulge [e.g., *Sergeev et al.*, 2004; *Henderson*, 2009]. If multiple and near-simultaneous BBFs occurred across the region encompassed by the SCW during this event, then the ionospheric current patterns may become more complex. In this particular event, up to 17 flow bursts (assuming two current sheets per BBF) would need to be present within an 8 min window within a region spanning 3 h of MLT (21–00 MLT) and produce multiple, overlapping and highly complex FAC signatures. Alternatively, the small-scale FAC associated with flow bursts may be more complicated than previously observed or modeled in the manner that *Sergeev et al.* [2004] predict, allowing for multiple current sheets of one sense or another. The lack of auroral data or magnetotail observations of plasma flows means that we cannot conclusively rule out BBFs as the source of the azimuthal structure; however, our observations provide a significant challenge to studies that conclude that BBFs can produce the small-scale current structure of the SCW. Further studies that include simultaneous auroral and magnetotail observations in addition to Cluster AAR passes are required to explore this.

Azimuthally periodic auroral forms are commonly seen on equatorward arcs prior to substorm breakup [*Murphree et al.*, 1994; *Elphinstone et al.*, 1995; *Donovan et al.*, 2007, 2008; *Liang et al.*, 2008; *Sakaguchi et al.*, 2009; *Henderson*, 2009; *Rae et al.*, 2009a, 2009b] and undulations in the poleward arc have been reported during a substorm recovery phase [*Motoba et al.*, 2012a]. The equatorward arc forms are observed to brighten and expand prior to the auroral breakup, which has been associated with various instabilities in the inner magnetosphere [*Rae et al.*, 2010]. Since most of the studies of these forms have concentrated on substorm onset mechanisms, these forms were not traced further into the expansion phase, so their continued existence is unclear. In one case study, *Elphinstone et al.* [1995] showed these forms were still observed at the equatorward edge of the auroral oval after the appearance of the substorm bulge. In another case study, *Henderson* [2009] showed that spatially periodic forms corresponding at substorm onset evolved into east-west aligned features in the equatorward arc and subsequently auroral streamers projected into the auroral bulge from the poleward arc. In contrast, *Motoba et al.* [2012a] showed no evidence of spatially periodic forms prior to the substorm but do show undulations in the poleward arc associated with field-aligned current signatures in the magnetosphere. All of these forms have ionospheric wavelength scales of the order of 100 km and are reported to travel eastward, similar to our interpretation of the observations of the current sheets within the substorm current wedge. This may suggest that onset instabilities in the inner magnetosphere impose some structure on the subsequent substorm current wedge that persists throughout the substorm even after the instability has ceased as opposed to the distant driving of a number >30 current sheets. However, scale size alone cannot be used to distinguish between structure imposed by an earlier instability and BBF activity, given that a 1 *R*_*E*_ wide BBF at 15 *R*_*E*_ downtail has maps to ∼100 km in the ionosphere. Further observations are needed to examine the link between preonset auroral forms, substorm current wedge substructure, and late-substorm auroral perturbations.

In this study, we have interpreted the magnetic field perturbations as being related to field-aligned current sheets that drifted dawnward and varied between the two spacecraft crossings. The magnetic field data from Cluster 4 and 1 could alternatively be interpreted as being due to wave activity, particularly in the net downward current region. In the upward current region, the electron distributions show evidence of quasi-static acceleration processes at both Cluster 4 and Cluster 1 and not the broadband acceleration associated with inertial Alfvén waves [*Chaston et al.*, 2007; *Lysak and Song*, 2011; *Marklund et al.*, 2011], strongly suggesting that the observations are of drifting current sheets, as opposed to being due primarily to Alfvén waves. However, we note that the net downward current region may be comprised of both quasi-static and Alfvénic signatures (c.f. Figures 4d and 5d). While Alfvén waves in the net downward current (if present) may be related to BBF activity [e.g., *Kepko et al.*, 2001; *Murphy et al.*, 2011], the quasi-static FAC signatures in the net upward current region cannot be explained by the current BBF framework, as discussed above. Given that the currents observed are relatively stationary between the two spacecraft crossings, we do not consider that any waves present significantly alter our conclusions.

## 4. Conclusions

Using a combination of multispacecraft observations from Cluster 1 and 4 during an AAR perigee pass, along with ground-based magnetometer and optical auroral data, we have provided new insights into the structure and temporal evolution of the substorm current wedge, revealing a plethora of north-south aligned current sheets at ∼100 km scales. Using data from Cluster 1 and Cluster 4 as they crossed auroral latitudes at 4000–7000 km, along with ground-based magnetometer and optical data, we have shown that the substorm current wedge consists of a number of upward and downward current sheets that, when spatially averaged, reduce to the simple line current model of the substorm current wedge of *McPherron et al.* [1973]. During the two spacecraft crossings, we identified 34 and 26 current sheets in the data from Cluster 1 and Cluster 4, respectively. These sheets had widths of up to 300 km, with a peak width of 100 km and tended to be aligned more north-south than east-west, in contrast to what one might expect for auroral current sheets but in keeping with the common observation of poleward boundary intensifications following substorm onset. Our observations challenge existing models which describe the substorm current wedge as single or dual current loops bracketing a region of dipolar field lines in the magnetotail [*McPherron et al.*, 1973; *Sergeev et al.*, 2011].

The widths, strengths, and structure of the FAC densities observed differed from the expected pattern of FACs associated with BBFs [*Forsyth et al.*, 2008] and auroral streamers [*Amm et al.*, 1999; *Nakamura et al.*, 2005; *Juusola et al.*, 2009], suggesting that the structure of the SCW is inconsistent with the proposed framework of the SCW being formed through a series of wedgelets [*Zhang et al.*, 2011; *Lyons et al.*, 2012, 2013; *Birn and Hesse*, 2013; *Liu et al.*, 2013] associated with BBFs. Our observations of small-scale structuring of the SCW show the same spatial properties to substorm azimuthal auroral forms [e.g., *Elphinstone et al.*, 1995; *Henderson*, 2009; *Rae et al.*, 2009a; *Motoba et al.*, 2012b] that have been shown to be due to instabilities operating in the near-Earth magnetotail [e.g., *Rae et al.*, 2010]. Whether the SCW structuring observed in this event is due to an ongoing instability or a fingerprint left from the onset process itself is unclear and is of fundamental importance for the study of the generation mechanism behind the SCW.

## Appendix A Discussion of Determining FAC From Single Spacecraft Magnetometer Data

Estimates of the field-aligned current densities flowing in a region can be made using the magnetic field data from a single spacecraft by assuming that perturbations in the magnetic field data are spatial rather than temporal and result from current sheets that are infinite in two dimensions. Under these assumptions, Ampére's law reduces to

(A1)

where *j* is the field-aligned current density, *Δ**B*_⊥_/d*t* is the temporal derivative of residual magnetic field perpendicular to both the background magnetic field and the spacecraft velocity vector, *v*_⊥sc_ is the spacecraft velocity perpendicular to the background field, and *θ* is the angle between the current sheet normal and the spacecraft velocity vector perpendicular to the background magnetic field [*Frey et al.*, 1998; *Peria et al.*, 2000]. In the case of a moving current sheet, *v*_⊥sc_ becomes the relative velocity between the current sheet and the spacecraft [after *Marchaudon et al.*, 2006]. *Forsyth et al.* [2012] showed that current densities determined by this technique from the FGM data and from the electron moments from the PEACE instrument on Cluster from a crossing of the auroral acceleration region were in agreement, particularly in the upward current region, providing an independent verification of this technique.

Spacecraft are unlikely to make normal crossings of the current sheets in the auroral region, thus determining *θ* is important. In order to calculate *θ*, we initially assume *θ*=0 and from this we identify individual current sheets. Minimum variance analysis (MVA) [*Sonnerup and Cahill Jr*., 1967; *Sonnerup and Scheible*, 1998] is then performed on the residual magnetic field in each of these sheets. Since we have removed the background field and have FAC, the minimum variance direction points in the direction of the current and the maximum variance direction points along the current sheet, perpendicular to current direction. From this, we can then determine the orientation of the current sheet and the angle at which the spacecraft crosses it (*θ*).

Determining the direction of the background field is also critical to this analysis. Empirical models, such as the *Tsyganenko and Stern* [1996] magnetic field model, are constructed based on the averages values of the observed magnetic field thus may not represent the true background field on a case-by-case basis. Filtering the data, using a running mean or a high-pass filter, may inadvertently remove some important perturbations. In this case study, we take the background field to be that from the *Tsyganenko and Stern* [1996] model, although a brief check showed that the results were similar for a 900 s high-pass filter.

In this study, we take the velocity of the auroral current sheets at the altitude of the Cluster spacecraft to be 1 km s^−1^, based on an approximate shift of the currents by 0.3 MLT in 120 s (Figures 4 and 5). Also, if *θ*>60°, we set *θ*=0 [after *Peria et al.*, 2000] in order to prevent exceptionally large currents which may not be well defined by the infinite current sheet model. This allows perturbations in the magnetic fields between the spacecraft to still be compared.

## Supporting Information

• Readme

• Animation S1
