# Supplementary material for: In situ spatiotemporal measurements of the detailed azimuthal substructure of the substorm current wedge
Source: J Geophys Res Space Phys. 2014 Feb 12;119(2):927–46. doi: 10.1002/2013JA019302 (PMC4497475; doi:10.1002/2013JA019302)
Supplement: Supplementary file 1 — • Readme [file jgra0119-0927-SD1.docx]

Auxiliary Material for

In-situ spatio-temporal measurements of the detailed azimuthal substructure of the substorm current wedge

C. Forsyth^1^, A.N. Fazakerley^1^, I .J. Rae,^1^, C.E.J. Watt^2^, K. Murphy^3^, J.A.Wild^4^, T. Karlsson^5^, R. Mutel^6^, C.J. Owen^1^, R. Ergun^7^, A. Masson^8^, E. Donovan^9^, H.U. Frey^10^, J. Matzka^11^, C. Stolle^11,12^, Y. Zhang^13^

1) Mullard Space Science Laboratory, UCL, Dorking, Surrey, UK
2) Dept. of Meteorology, University ofReading, Reading, UK
3) University of Alberta, Edmonton, Alberta, Canada
4) Lancaster University, Lancaster, UK
5) Royal Institute of Technology, Stockholm, Sweden
6) University of Iowa, Iowa City, Iowa, USA
7) LASP, University of Colorado, Boulder, Colorado, USA
8) ESA/ESTEC, Noordwijk, Netherlands
 9) University of Calgary, Calgary, Canada
10) University of California, Berkeley, California, USA
11) DTU
12) GFZ, German Centre for Geoscience, Potsdam, Germany
13) John Hopkins University Applied Physics Laboratory, USA

Journal of Geophysical Research, Space Physics, 2013

Introduction

This animation shows data from the DMSP SSUSI ultraviolet imager, the THEMIS white-light all-sky imager (ASI) at Rankin Inlet and from the FluxGate Magnetometer on Cluster 1, 2 and 4 between 02:20 UT and 03:00 UT on 15 January 2010. Individual frames of this animation are shown in Fig, 3 of the paper. The data are presented in magnetic local time and invariant latitude coordinates.

1. ms01.mp4 Animation of data from the THEMIS ASI at Rankin Inlet, the SSUSI instrument of DMSP F16, the magnetic foot points of Cluster 1, 2 and 4, and the magnetic field gradients perpendicular to the spacecraft trajectory and the Tsyganenko & Stern (1996) magnetic field model. Magnetic field gradients from Cluster 2 and Cluster 4 point upwards and magnetic field gradients from Cluster 1 point downwards. The magnetic field gradients are color coded with respect to the direction of a field-aligned current associated with those gradients; red indicates upward current, blue indicates downward current.
